# Editorial: Mechanisms underlying psychological resilience and post-traumatic growth

**DOI:** 10.3389/fpsyg.2023.1230055

**Published:** 2023-06-28

**Authors:** Rosaura Gonzalez-Mendez, Hipólito Marrero, Vincenzo Romei

**Affiliations:** ^1^Departamento de Psicología Cognitiva, Social y Organizacional, San Cristóbal de La Laguna, Spain; ^2^Instituto Universitario de Neurociencias (IUNE), San Cristóbal de La Laguna, Spain; ^3^Centro Studi e Ricerche in Neuroscienze Cognitive, Dipartimento di Psicologia “Renzo Canestrari” Università di Bologna, Cesena, Italy; ^4^Facultad de Lenguas y Educación, Universidad Antonio de Nebrija, Madrid, Spain

**Keywords:** resilience, post-traumatic growth, affective mechanisms, motivational mechanisms, cognitive styles, neural activity, health, individual differences

Researchers have long been fascinated by the ability of some people to overcome adversity. Over the last decades, psychological resilience has been examined across numerous populations and domains, providing evidence that the human ability to adapt to adverse conditions is much more frequent than initially thought (Masten, 2001). Resilient people are expected to maintain relatively stable and healthy levels of functioning, recover better after a temporary decline, or thrive beyond the pre-trauma level of functioning (Fisher et al., 2019).

Psychological resilience is increasingly seen as a dynamic process that operates at multiple levels (Bonanno et al., 2015; Denckla et al., 2020; Masten et al., 2021). Understanding the mechanisms underlying this process can help identify effective ways to promote resilience both in the general population and in vulnerable people. The papers in this issue explore potential affective and cognitive mechanisms that can account for psychological resilience in relation to several types of adversity (e.g., COVID-19 emergency), and in a variety of contexts including educational, organizational, etc. Particular attention is dedicated to the consequences of post-traumatic growth (PTG) and its psychological underlying mechanisms in health and wellbeing.

According to Southwick et al. (2014), moving forward and struggling are important components of psychological resilience. This idea guides several of the studies contributing to this Research Topic, which directly or indirectly point to motivational mechanisms to account for resilience.

While research has consistently shown that experiences of peer victimization may have long lasting negative consequences on health and academic achievement, few studies have examined the resilience and PTG of those students who experienced bullying before entering college (Andreou et al., 2021; Yubero et al., 2023). Ravelo et al. examine the motivational mechanisms underlying PTG in this population. After analyzing the role of different motivational orientations as potential mediators, the results support that regulatory focus on promotion (i.e., focusing attention on life changes and challenges as opportunities) mediates between resilient coping and thriving. Instead of being a distal dispositional trait, regulatory focus is considered a proximal motivational process (Lanaj et al., 2012). This means that it may be enhanced through training cognitive strategies, such as resilient coping.

Pathways to resilience may be multiple and sometimes unexpected (Bonanno, 2004). In this regard, Rohner et al. explore how later life prosocial behavior is related to positive adaptation to experiences of childhood adversity. They identify three themes that are common to two groups of survivors, and that are positively related to prosocial behavior. Enhanced empathy due to their experiences, self-identity linked to their choice of a social/caring profession, and amelioration through meaning-making and sense of purpose. These findings are consistent with previous research that indicate how life narratives are associated with resilience by redefining experiences and one's own identity (Gonzalez-Mendez et al., 2022; Ramasubramanian et al., 2022). However, they also point to motivational processes linked to the need for a sense of purpose and meaning from the adversities.

Courage is defined as an approaching behavior despite the experience of fear, which has been shown to be negatively associated with stress in high risk occupations. However, knowledge about the mechanisms by which courage positively regulates stress is still scarce. Wang et al. analyze the mediating role of individual motivational differences in Behavioral Activation System (BAS) and Behavioral Inhibition System (BIS) (Gray, 1991; Carver and White, 1994) on the relationship between courage and stress. The results show that courage may decrease stress by reducing the activity of BIS and fun-seeking. They suggest that the need to inhibit the latter may be due to its association with impulsiveness, which interferes with courage during actions that require planning.

The mediating effect of mood states on the relationship between psychological resilience and emotional stability are explored by Han and Wang in high school students in China during the COVID-19 pandemic. They find that psychological resilience directly relates to emotional stability and that positive and negative moods mediate on this relationship. In contrast to previous research, the mediating effect of negative mood was greater than positive mood. As the association of positive mood for enhancing the effect of resilience in emotional stability was found prior to the COVID-19 emergency, they speculate that the greater role of negative mood is associated with the psychological consequences of the pandemic.

Beliefs have been shown to be associated with PTG (Vazquez et al., 2021), and two studies address the role of different cognitive frameworks in the resilience process. Implicit theories (or mindsets) refer to beliefs that people hold regarding whether abilities are fixed (entity theory) or variable (incremental theory), which have shown to be relevant for predicting psychological resilience (Yeager and Dweck, 2012; Boullion et al., 2021). However, the mechanisms that contribute to explaining the relationship between both factors are poorly understood. Tang et al. analyze the chain mediating role of grit and meaning in life on the relationship between implicit theories and resilience. In addition to confirming the significant weight of incremental theory in predicting the resilience of Chinese nurses, their results shed light on this link by supporting the partial mediating role of grit and meaning in life.

Predictive coding theories refer to the ability to make decisions by integrating new sensory evidence into internal models of reality. Depending on the relative weight given to sensory evidence vs. internal model in decision-making strategy, interindividual differences in evidence-based vs. model-based beliefs have been highlighted (Tarasi et al., 2022). Tarasi et al. investigate how personality factors can predict attitudes that facilitate resilience, such as antivax attitudes during the COVID-19 pandemic. They show that evidence-based beliefs (associated with autistic traits) favor positive attitudes toward vaccination, whereas model-based beliefs (associated with schizotypal traits) favor antivax attitudes. Importantly, while this dichotomy increases with age, its impact is mitigated by education level, which favors psychological resilience. Thus, evidence-based (autistic) more than model-based (schizotypal) traits promote the adoption of beliefs supporting psychological resilience, which are mediated by education. Tarasi et al. also point to brain oscillatory activity in the alpha frequency band (Di Gregorio et al., 2022), as a physiological attribute that can predict evidence-based beliefs, making it easier to adopt more resilient, adaptive perspectives.

Psychological resilience is no longer considered an end-state or binary outcome (Khanlou and Wray, 2014). Hence, longitudinal studies are appropriate for detecting the changes that occur after a traumatic event. Gil-González et al. explore PTG after diagnosis and adaptation to multiple sclerosis (MS). Their results show significant positive intrapsychic changes of MS patients over a 36-month follow-up period up to 12 years from diagnosis. In addition, they find comparable levels of PTG in both patients and caregivers, and suggest interdependent cognitive processes, such as acceptance and meaning making as psychological mechanisms involved in PTG.

Some approaches to psychological interventions have already been implemented aiming at favoring PTG. In a systematic review and meta-analysis, Pierce et al. explore the impact that three different types of interventions (namely cognitive processing therapy, eye movement desensitization and reprocessing, and prolonged exposure therapy) have on neural activity underlying the phenomenon of PTG for adult trauma survivors. They found promising confirmations with a robust effect of all these approaches and a stronger impact of eye movement desensitization and reprocessing on PTG and brain functions.

Exposure to adverse experiences has been shown to have negative consequences on health and wellbeing. However, research on resilience offers a way to anticipate the appearance of maladaptive responses by strengthening protective mechanisms. In this Research Topic there are relevant contributions that shed light on the affective and cognitive mechanisms underlying resilience. The studies gathered below show how these mechanisms help people deploy psychological resources and thrive in the face of different types of adversities and social contexts.

## Author contributions

All authors listed have made a substantial, direct, and intellectual contribution to the work and approved it for publication.
